# Genomic Characterisation of a Novel Avipoxvirus Isolated from an Endangered Northern Royal Albatross (*Diomedea sanfordi*)

**DOI:** 10.3390/pathogens10050575

**Published:** 2021-05-09

**Authors:** Subir Sarker, Ajani Athukorala, Tadiwa Nyandowe, Timothy R. Bowden, David B. Boyle

**Affiliations:** 1 Department of Physiology, Anatomy and Microbiology, School of Life Sciences, La Trobe University, Melbourne, VIC 3086, Australia; a.athukorala@latrobe.edu.au (A.A.); 19918447@students.latrobe.edu.au (T.N.); 2 CSIRO Livestock Industries, Australian Animal Health Laboratory, Geelong, VIC 3220, Australia; timothy.bowden@csiro.au (T.R.B.); davidboyle48@gmail.com (D.B.B.)

**Keywords:** avipoxvirus, complete genome, endangered, evolution, northern royal albatross

## Abstract

Marine bird populations have been declining globally with the factors driving this decline not fully understood. Viral diseases, including those caused by poxviruses, are a concern for endangered seabird species. In this study we have characterised a novel avipoxvirus, tentatively designated albatrosspox virus (ALPV), isolated from a skin lesion of an endangered New Zealand northern royal albatross (*Diomedea sanfordi*). The ALPV genome was 351.9 kbp in length and contained 336 predicted genes, seven of which were determined to be unique. The highest number of genes (313) in the ALPV genome were homologs of those in shearwaterpox virus 2 (SWPV2), while a further 10 were homologs to canarypox virus (CNPV) and an additional six to shearwaterpox virus 1 (SWPV1). Phylogenetic analyses positioned the ALPV genome within a distinct subclade comprising recently isolated avipoxvirus genome sequences from shearwater, penguin and passerine bird species. This is the first reported genome sequence of ALPV from a northern royal albatross and will help to track the evolution of avipoxvirus infections in this endangered species.

## 1. Introduction

Marine bird populations have been declining globally [1] with the sustainability of the albatrosses (family *Diomedeidae*) and large petrels (*Macronectes* and *Procellaria* spp.) being of particular concern [2,3,4]. This group includes some of the world’s most endangered bird species, with rapidly decreasing populations and their conservation status markedly deteriorating in recent years [5,6]. The northern royal albatross (*Diomedea sanfordi*), which is one of the largest seabirds in the world, is categorised as an “endangered” species under the International Union for Conservation of Nature (IUCN) Red List and is ranked as Category B for conservation priority [7]. The northern royal albatrosses range widely throughout the Southern Ocean, though rarely into Antarctic waters. The breeding range is restricted to the Chatham Islands and Taiaroa Head on the Otago Peninsula, Dunedin, New Zealand. The total breeding population in the Chatham Islands colonies (99% of the total) is estimated at approximately 6500–7000 pairs, which equates to a total population of 17,000 mature individuals [8]. Northern royal albatrosses are normally solitary foragers, but they may congregate at food sources at sea. Most of their food is thought to be obtained by seizing dead or dying prey from the surface and also by scavenging discards and offal from fishing boats. Breeding birds forage over the continental shelves to shelf edges in New Zealand waters. Non-breeding and young birds can be found anywhere in the Southern Ocean throughout the year, with the main wintering areas off the coasts of southern South America [8].

Human activities such as fisheries and pollution have been documented as threats for incidental mortality of these species [7,9,10,11,12,13]. Invasive alien species, degradation or loss of nesting habitats, storms and flooding, and marine pollution or plastic ingestion are also significant factors in population declines [6,7]. Infectious diseases, including those caused by avipoxviruses, have been identified as an important risk factor in the conservation of small and endangered bird populations, including albatrosses [14,15,16,17,18,19]. The impact of the introduction of avipoxviruses has been severe for the avifauna of various archipelagos [20]. For example, the emergence of an avipoxvirus with a high prevalence (88%) in Hawaiian Laysan albatrosses (*Phoebastria immutabilis*) enabled one of the first detailed studies of the epidemiology and population-level impact of the disease in these seabirds [21].

Avipoxviruses are large, double-stranded DNA (dsDNA) viruses comprising the genus *Avipoxvirus.* They occur worldwide and are known to infect a large number of wild and domestic avian species across 76 families and 20 orders [22,23,24]. The behaviour of wild birds allows avian poxviruses to reach new hosts through bird migration, species introductions, and habitat change. Avipoxviruses have been identified as an important risk factor in the conservation of endangered bird populations [19,25]. In affected birds, avipoxvirus infection can cause two different forms of disease, defined as cutaneous or diphtheritic. The cutaneous form is characterised by proliferative ‘wart-like’ lesions that commonly develop on unfeathered body areas, including the eyes, feet, legs, face and around the beak. The less common diphtheritic form is characterised by soft and yellowish cankers and proliferative lesions on the mucous membranes of the upper alimentary and respiratory tracts [23,26,27].

Little is known about the effects of poxviruses on some bird taxa, particularly for seabird species including the northern royal albatross (*D. sanfordi*). The aim of the present study was to characterise the genome sequence of a novel poxvirus, which was isolated from a skin lesion that was collected in 1997 from an endangered northern royal albatross on the Otago Peninsula, near Dunedin, on the South Island of New Zealand.

## 2. Results

### 2.1. Genome of ALPV

The complete genome of ALPV was assembled into a contiguous sequence of linear double-stranded DNA 351,909 bp in length (the second-largest avipoxvirus genome so far characterised) and submitted to GenBank under accession number MW365933. Like many other avipoxviruses [25,28,29], the ALPV genome contained a well-conserved central coding region surrounded by two identical inverted terminal repeat (ITR) regions, comprising 4069 bp each (coordinates 1–4069 sense and 347,841–351,909 antisense orientation). The nucleotide composition of the ALPV genome was A + T rich (69.9%), which was in agreement with other avipoxviruses isolated from yellow-eyed penguin [19], shearwater [25] and passerine bird species [30,31] (Table 1). The ALPV genome showed the highest nucleotide identities with penguinpox virus 2 (PEPV2, GenBank accession no. MW296038) (98.92%), followed by shearwaterpox virus 2 (SWPV2, GenBank accession no. KX857216) (95.75%), canarypox virus (CNPV, GenBank accession no. AY318871) (92.71%) and mudlarkpox virus (MLPV, GenBank accession no. MK903864) (88.47%) (Table 1).

### 2.2. Genome Annotation and Comparative Analyses of ALPV

**Figure 1:**
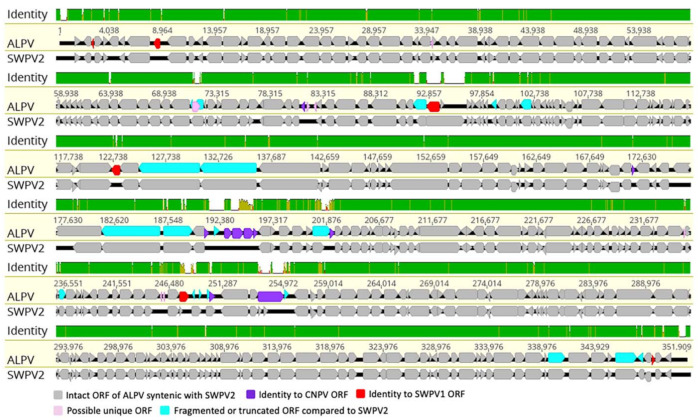
Comparative genomic illustration of the novel ALPV. A sequence alignment using MAFFT in Geneious (version 10.2.2) was performed to compare ORFs between albatrosspox virus (ALPV, GenBank accession no. MW365933) and shearwaterpox virus 2 (SWPV2, GenBank accession no. KX857215). The arrows symbolise genes and open reading frames (ORFs), with orientation indicating their direction of transcription. Each gene or ORF is colour coded, as indicated by the key in the legend. The top graph represents the mean pairwise sequence identity over all pairs in the column between ALPV and SWPV2 (green: 100% identity; mustard: ≥30% and <100% identity; red: <30% identity).

The ALPV genome encoded 336 putative genes, 45 to 1936 amino acids in length, that have been numbered from left to right (Figure 1 and Table 2). Among them, four ORFs were located within the inverted terminal repeats (ITRs) and were therefore present as diploid copies. Comparative analysis of the predicted ORF sequences was performed, and a significant number of ORFs (329) were found to be homologs with other chordopoxvirus (ChPV) gene products (Table 2). Among these conserved ChPV gene products, the highest number of protein-coding genes (313) in ALPV were homologs to the recently isolated SWPV2 [25]. The remaining ten gene products (ALPV-079, -155, -163, -165, -166, -167, -168, -175, -233 and -236) were homologous to ORFs of CNPV, and a further six gene products (ALPV-003, -009, -090, -127, -229 and -334) were homologs to SWPV1 (Table 2). All conserved genes of ALPV showed the highest sequence similarity to homologs of avipoxviruses isolated from Pacific shearwater, canary and yellow-eyed penguin bird species, implying a common evolutionary history [19,25,32]. In comparison to SWPV2, two gene products (SWPV2-121 and -213) were absent from the ALPV genome, and a further nineteen genes were predicted to be truncated/fragmented (Figure 1 and Table 2). In comparison to vaccinia virus strain Copenhagen (VACV-Cop), 138 ORFs of ALPV showed homology to VACV-Cop and the sequence identities ranging from 20.9–76.7% (Table 2).

Interestingly, ALPV contained seven predicted protein-coding genes (ORF030, -067, -080, -081, -213, -226 and -227) that were not present in any other characterised poxvirus genomes, nor did they match any sequences in the NR protein database using BLASTX and BLASTP; these unique ORFs encoded proteins of 51 to 89 amino acids in length (Table 2). Furthermore, four of these unique protein-coding genes (ALPV-ORF030, -213, -226 and -227) were predicted to contain a single transmembrane helix (TMH) using the software packages employed in this study (Table 2). However, we did not find any known motif, nor significant homology with known proteins, for the unique ORFs encoded in the ALPV genome when using the Phyre2, HHpred and SWISS-MODEL, which might be due to the lack of closely related structures in the database.

**Figure 2:**
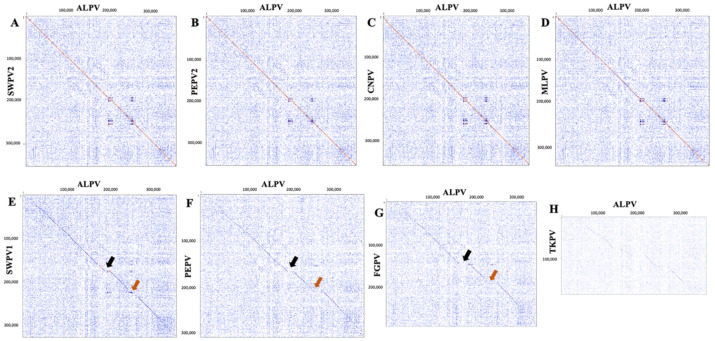
Dot plots of the ALPV genome (*x*-axis) vs. other poxvirus genomes (*y*-axis). (**A**) ALPV vs SWPV2, (**B**) ALPV vs PEPV2, (**C**) ALPV vs CNPV, (**D**) ALPV vs MLPV, (**E**) ALPV vs SWPV1, (**F**) ALPV vs PEPV, (**G**) ALPV vs FGPV and (**H**) ALPV vs TKPV (refer to Table 2 for virus details and GenBank accession numbers). The Classic colour scheme was chosen in Geneious (version 10.2.2) for the dot plot lines according to the length of the match, from blue for short matches to red for matches over 100 bp long. Window size = 12.

Comparison of the ALPV genome to that of other avipoxviruses was performed using dot plot analyses. The ALPV genome was shown to be highly syntenic with SWPV2, PEPV2, CNPV and MLPV (Figure 2A–D) and demonstrated significant differences compared to SWPV1, PEPV, FGPV and TKPV (black and orange arrows, Figure 2E–H).

### 2.3. Evolutionary Relationships of ALPV

**Figure 3:**
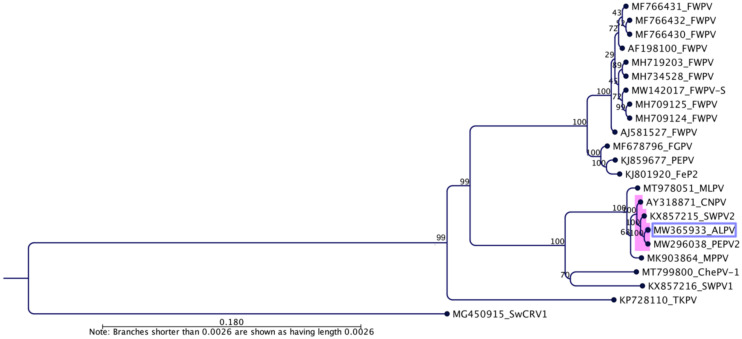
Phylogenetic relationships between ALPV and other chordopoxviruses. A maximum likelihood (ML) tree was constructed from multiple alignments of the concatenated amino acid sequences of the selected nine poxvirus core proteins using CLC Genomic Workbench (version 9.5.4, CLC bio, a QIAGEN Company, Prismet, Aarhus C, Denmark). The numbers on the left show bootstrap values as percentages. The ML tree is displayed as a phylogram. The labels at branch tips refer to original ChPV GenBank accession numbers followed by abbreviated species names. Saltwater crocodilepox virus (SwCRV1) [36] was used as an outgroup. The position of the novel ALPV is highlighted using a purple box and the subclade relevant to ALPV is shown with pink shading.

Phylogenetic analysis using concatenated amino acid sequences of the selected nine core poxvirus proteins supported the inclusion of the newly assembled ALPV in the genus *Avipoxvirus*. In the maximum likelihood (ML) tree, ALPV was located within a sub-clade comprising SWPV2, PEPV2 and CNPV with strong bootstrap support (100%) (Figure 3), suggesting that it may represent an ancient evolutionary lineage within the genus. Using the same set of concatenated protein sequences, we found that the maximum inter-lineage sequence identity values were 100% among ALPV, SWPV2 and PEPV2, which mirrored the phylogenetic position of this novel avipoxvirus sequenced from an endangered northern royal albatross. A large number of poxviruses were positioned in the phylogenetic tree when we used partial nucleotide sequences of the DNA polymerase gene (Supplementary Figure S1) and p4b gene (Supplementary Figure S2). We discovered that several other avipoxviruses were represented within the ALPV, PEPV2, CNPV, SWPV2, MPPV and MLPV clade. This included a poxvirus isolated from a common bullfinch (*Pyrrhula pyrrhula*) in Belgium [35] and a northern harrier (*Circus cyaneus*) in Spain [35], which is almost identical to ALPV within this relatively small fragment of the genome.

## 3. Discussion

This study presents the characterisation of the complete genome sequence of a novel avipoxvirus, ALPV, isolated from cutaneous pox lesions in a juvenile northern royal albatross. In addition to the highest number of genes being homologous to SWPV2, a further ten were homologs to CNPV and six to SWPV1. An additional seven genes were not present in any other known poxvirus, nor did they match any sequences in the NR protein database. Given this genome structure, gene content, genome nucleotide similarities and phylogenetic relationships, the authors postulate that the ALPV genome is most closely related to avipoxviruses isolated from shearwater, penguin and canary bird species.

It has been reported that an avipoxvirus may have caused the death of some northern royal albatrosses in 1997 [37]. However, as far as we are aware, there have been no further scientific studies investigating the epidemiology and characteristics of avipoxviruses circulating in this population. Moreover, avipoxviruses have been reported to be a significant cause of chick mortality in several other albatross species. For instance, an avipoxvirus spread by bird fleas has caused high chick mortalities in some seasons within colonies of shy albatrosses (*Thalassarche cauta*) in Tasmania [38] and may act as a potential threat to adults and chicks of Buller’s albatross (*Thalassarche bulleri*) [37]. An avipoxvirus has also been described as a key threat for the rapid population decline of a critically endangered seabird, the waved Albatross (*Phoebastria irrorata*), in the Galápagos Islands, Ecuador [39]. Given the conservation status of the northern royal albatross, it would be important to improve understanding of the epidemiology, transmission and genetic diversity of circulating avipoxviruses, including ALPV, and the threat that they pose to this and other albatross species.

Identifying the transmission mode is essential to characterise incidence, ecology, and effective control of disease in wild populations. However, it is not yet known how this avipoxvirus is transmitted. Mechanical transmission by biting arthropods is thought to play a role in the transmission of avipoxviruses within wild bird populations. Ticks, fleas [40], hippoboscid flies [41], and mosquitos [20,42] are all potential mechanical vectors. It would therefore seem likely that, as for other avipoxviruses, transmission of ALPV in the northern royal albatross is also mediated by insect vectors. Moreover, poxvirus infection can also occur through ingestion, parenteral inoculation, or droplet or aerosol exposure to mucous membranes or broken skin. Some poxviruses can be transmitted by fomites (inanimate objects) [43]. For example, studies have revealed that sheeppox and goatpox viruses are predominantly transmitted via aerosols [44]; whereas poxviruses from the genus *Parapoxvirus* can pass from one animal to another through direct or indirect contact. However, unfortunately, there are no available studies addressing whether closely related avipoxviruses employ similar or different routes of spread.

There are a number of factors that threaten the populations of large sea birds, particularly albatrosses. Amongst these are longline fishing, climate change and diseases such as those caused by avipoxviruses. It is well-established in the literature that avipoxviruses are mechanically transmitted by biting insects. Although yet to be confirmed, it is therefore expected that this will also be the case for ALPV.

## 4. Materials and Methods

### 4.1. Sampling and Virus Isolation

Cutaneous pox lesions were collected from an endangered juvenile northern royal albatross (*Diomedea sanfordi*), located on the Otago Peninsula, near Dunedin, on the South Island of New Zealand. Sampling was conducted in March 1997 by Wallaceville Animal Research Centre, New Zealand, and lesions sent to the Australian Animal Health Laboratory, Geelong, Victoria, Australia (sample ID: SL 08/05/1997). Virus isolation was undertaken by homogenisation of the tissue samples (~10% *w*/*v*) in the presence of antibiotics. This material was then inoculated onto the chorioallantoic membranes (CAMs) of 10 to 12 day old embryonated chicken eggs. The CAMs were harvested 3 to 5 days later and examined for the presence of pock lesions. The infected CAMs were similarly homogenised and passaged onto fresh CAMs. Similarly, the homogenised tissue samples were inoculated onto monolayers of chicken embryo skin cells and examined for 7 to 10 days for the development of cytopathic effect. Additional passages were undertaken with frozen and thawed tissue culture cells inoculated onto fresh chicken embryo skin cells. All passages were stored frozen at −80 °C.

### 4.2. DNA Extraction and Sequencing

Infected cell culture pellets were digested with DNAase and RNAase, and then with trypsin. Released virus was pelleted through a 36% sucrose cushion for 80 min at 20,000 rpm. Poxvirus cores were released from the pelleted virus with 1% Triton X100 and mercaptoethanol by incubation for 10 min on ice. The released cores were pelleted through a 36% sucrose cushion and the viral DNA released by Proteinase K/RNAase digestion followed by phenol/chloroform extraction and ethanol precipitation, as reported previously [45,46]. Sequencing was undertaken using TruSeq (Illumina) protocols and standard multiplex adaptors available in March 2011. A paired-end 100-base-read protocol was used for sequencing on an Illumina GAIIx instrument using a previously established protocol [47].

### 4.3. Genome Assembly and Annotation

The resulting 3,343,202 paired-end raw sequence reads were used to assemble the complete genome of ALPV as described previously [25,31,48,49] using CLC Genomics Workbench (version 9.5.4, CLC bio, a QIAGEN Company, Prismet, Aarhus C, Denmark) and Geneious (version 10.2.2, Biomatters, New Zealand). Briefly, the sequences were processed to remove Illumina adapters, low quality reads and ambiguous bases. Trimmed sequence reads were mapped against the chicken genome (*Gallus gallus*, GenBank accession number NC_006088) to remove likely host DNA contamination. In addition, reads were further mapped to *Escherichia coli* bacterial genomic sequence (GenBank accession no. U00096) to remove possible bacterial contamination. Unmapped reads were used as input data for *de novo* assembly using CLC Genomics Workbench (version 9.5.4). This resulted in the generation of a 351,909 bp genome. Clean raw reads (1.15 million) were mapped back to the assembled ALPV genome and resulted in an average coverage of 136.33x. The genome was annotated according to the previously published protocol [19] using Geneious software (version 10.2.2, Biomatters, Auckland, New Zealand). Open reading frames (ORFs) longer than 50 amino acids, with a methionine start codon (ATG) and minimal overlap with other ORFs (not exceeding 50% of one of the genes), were selected and annotated. ORFs shorter than 50 amino acids that had been previously annotated in other poxvirus genomes were also included. Similarity BLAST searches were performed on the predicted ORFs and were annotated as potential genes if predicted ORFs showed significant sequence similarity to known viral or cellular genes (BLAST E value ≤ e−5) [50]. Additional BLAST searches were performed on the predicted ORFs of ALPV against VACV-Cop [51].

To predict the function of unique ORFs tentatively identified in this study, the derived protein sequence of each ORF was searched by multiple applications to identify conserved domains or motifs. Transmembrane helices were searched using the TMHMM package (version 2.0) [52] and TMpred [53]. Additionally, searches for conserved secondary structure (HHpred) [54] and protein homologs using Phyre2 [55] were used to predict the function of unique ORFs identified in this study. To identify the likely promoter sequences of predicted unique ORFs of ALPV, a promoter motif search analysis was conducted using CLC Genomic Workbench (version 9.5.4), where vaccinia virus unique promoter sequences were used [51,56,57,58].

### 4.4. Comparative Genomics

Genomic features of the newly sequenced ALPV were visualised using Geneious (version 10.2.2). Sequence similarity percentages between representative ChPV and ALPV complete genome sequences were determined using tools available in Geneious (version 10.2.2). Dot plots were created based on the EMBOSS dottup program in Geneious software, with word size = 12 [59].

### 4.5. Phylogenetic Analyses

Phylogenetic analyses were performed using the novel ALPV genome sequence determined in this study, together with other selected ChPV genome sequences available in GenBank (Table 3). Nucleotide sequences of the partial DNA polymerase and partial p4b genes, as well as concatenated amino acid sequences of the selected nine poxvirus core proteins, were aligned as described previously [30] using the MAFTT L-INS-I algorithm implemented in Geneious (version 7.388) [60]. To determine the best-fit model to construct phylogenetic analyses, a model test was performed using CLC Genomics Workbench (version 9.5.4), which favoured a general-time-reversible model with gamma distribution rate variation and a proportion of invariable sites (GTR+G+I). Phylogenetic analyses for nucleotide sequences were performed using the GTR substitution model with 1000 bootstrap support in CLC Genomics Workbench (version 9.5.4), but the LG substitution model was chosen for concatenated amino acid sequences in Geneious (version 10.2.2).

## 5. Conclusions

This study reports the genomic characterisation of a novel avipoxvirus, ALPV, isolated from an endangered northern royal albatross. The ALPV genome sequence was sufficiently divergent from other known avipoxviruses to be considered a novel species within the genus *Avipoxvirus*, family *Poxviridae*. This discovery has enhanced our understanding of the pathogen landscape relevant to northern royal albatrosses in New Zealand. Obtaining and sequencing additional poxvirus isolates will also be important to further investigate the epidemiology, transmission, pathogenesis and host specificity of ALPV infections in this endangered bird species.

## Figures and Tables

**Table 1:** Comparative analysis of representative avipoxviruses and ALPV based on complete genome nucleotide sequences.

Avipoxviruses (Abbreviation)	GenBank Accession Numbers	Genome Identity (%)	Genome Length (kbp)	A + T Content (%)	Number of ORFs	References
Albatrosspox virus (ALPV)	MW365933		352	69.9	336	This study
Penguinpox virus 2 (PEPV2)	MW296038	98.92	350	69.9	327	[19]
Shearwaterpox virus 2 (SWPV2)	KX857215	95.75	351	69.8	312	[25]
Canarypox virus (CNPV)	AY318871	92.71	360	69.6	328	[32]
Mudlarkpox virus (MLPV)	MT978051	88.47	343	70.2	352	[30]
Magpiepox virus (MPPV)	MK903864	78.75	293	70.4	301	[31]
Shearwaterpox virus 1 (SWPV1)	KX857216	61.44	327	72.4	310	[25]
Penguinpox virus (PEPV)	KJ859677	49.83	307	70.5	285	[33]
Fowlpox virus (FWPV)	AF198100	48.89	289	69.1	260	[29]
Pigeonpox virus (FeP2)	KJ801920	47.54	282	70.5	271	[33]
Flamingopox virus (FGPV)	MF678796	46.51	293	70.5	285	[24]
Turkeypox virus (TKPV)	KP728110	33.39	189	70.2	171	[34]

**Table 2:** Albatrosspox virus (ALPV) genome annotations and comparative analysis of ORFs.

ALPV Synteny	ALPV Genome Coordinates	SWPV2 Synteny	ALPV AA Size	SWPV2 AA Size	SWPV2 BLAST Hits	ALPV AA Identity (%) Compared to Avipoxviruses	ALPV AA Identity (%) Compared to VACV-Cop	VACV BLAST Hits	Notes
ALPV-001	827-1342	SWPV2-001	171	171	SWPV2-001 hypothetical protein	100			identical to ALPV-336
ALPV-002	2271-1645	SWPV2-002	208	208	SWPV2-002 C-type lectin-like protein	100			identical to ALPV-335
ALPV-003	2553-2326		75			56.1			SWPV1-002 C-type lectin-like protein, identical to ALPV-334
ALPV-004	2679-3347	SWPV2-003	222	222	SWPV2-003 conserved hypothetical protein	100			identical to ALPV-333
ALPV-005	3464-3925	SWPV2-004	153	134	SWPV2-004 conserved hypothetical protein	86.9			identical to ALPV-332
ALPV-006	4899-4042	SWPV2-005	285	169	SWPV2-005 C-type lectin-like protein	46.9			
ALPV-007	5455-4946	SWPV2-005	169	169	SWPV2-005 C-type lectin-like protein	100			
ALPV-008	7809-5743	SWPV2-006	688	688	SWPV2-006 ankyrin repeat protein	100	23.9	B4R	
ALPV-009	8828-8184		214			43.1			SWPV1-006 ankyrin repeat protein
ALPV-010	11218-9458	SWPV2-007	586	586	SWPV2-007 ankyrin repeat protein	100	28.8	M1L	
ALPV-011	11483-12052	SWPV2-008	189	189	SWPV2-008 conserved hypothetical protein	100			
ALPV-012	12765-12259	SWPV2-009	168	168	SWPV2-009 conserved hypothetical protein	100			
ALPV-013	14555-13083	SWPV2-010	490	490	SWPV2-010 Ig-like domain protein	100			
ALPV-014	14719-16368	SWPV2-011	549	528	SWPV2-011 ankyrin repeat protein	96.2	28.7	M1L	
ALPV-015	16429-16935	SWPV2-012	168	168	SWPV2-012 C-type lectin-like protein	100	24.0	A40R	
ALPV-016	17039-18478	SWPV2-013	479	479	SWPV2-013 ankyrin repeat protein	100	26.7	B4R	
ALPV-017	19149-18577	SWPV2-014	190	190	SWPV2-014 IL-10-like protein	100			
ALPV-018	20576-19266	SWPV2-015	436	436	SWPV2-015 ankyrin repeat protein	100	27.5	M1L	
ALPV-019	20767-22026	SWPV2-016	419	419	SWPV2-016 ankyrin repeat protein	100	38.2	M1L	
ALPV-020	23769-22162	SWPV2-017	535	535	SWPV2-017 ankyrin repeat protein	100	21.5	B4R	
ALPV-021	24886-23810	SWPV2-018	358	358	SWPV2-018 putative serpin	100	27.9	C12L	
ALPV-022	26242-24968	SWPV2-019	424	424	SWPV2-019 vaccinia C4L/C10L-like protein	100-			
ALPV-023	26520-27056	SWPV2-020	178	178	SWPV2-020 hypothetical protein	100			
ALPV-024	28172-27270	SWPV2-021	300	300	SWPV2-021 alpha-SNAP-like protein	100			
ALPV-025	29443-28295	SWPV2-022	382	382	SWPV2-022 ankyrin repeat protein	100	22.0	C9L	
ALPV-026	31392-29512	SWPV2-023	626	626	SWPV2-023 ankyrin repeat protein	100			
ALPV-027	32608-31511	SWPV2-024	365	365	SWPV2-024 ankyrin repeat protein	100			
ALPV-028	33146-32718	SWPV2-025	142	142	SWPV2-025 C-type lectin-like protein	100			
ALPV-029	34224-33202	SWPV2-026	340	340	SWPV2-026 ankyrin repeat protein	100	26.8	B4R	
ALPV-030	34487-34287		66						hypothetical protein, unique to ALPV, containing a transmembrane helix
ALPV-031	34467-34826	SWPV2-027	119	119	SWPV2-027 hypothetical protein	100			
ALPV-032	35782-35054	SWPV2-028	242	242	SWPV2-028 Ig-like domain putative IFN-gamma binding protein	100			
ALPV-033	36598-35858	SWPV2-029	246	246	SWPV2-029 Ig-like domain protein	100			
ALPV-034	38673-36694	SWPV2-030	659	659	SWPV2-030 ankyrin repeat protein	100	23.5	K1L	
ALPV-035	39392-38991	SWPV2-031	133	133	SWPV2-031 C-type lectin-like protein	100			
ALPV-036	39776-39489	SWPV2-032	95	95	SWPV2-032 conserved hypothetical protein	100			
ALPV-037	39840-40379	SWPV2-033	179	179	SWPV2-033 conserved hypothetical protein	100			
ALPV-038	41625-40384	SWPV2-034	413	413	SWPV2-034 vaccinia C4L/C10L-like protein	99.8	24.3	C10L	
ALPV-039	41743-42726	SWPV2-035	327	327	SWPV2-035 G protein-coupled receptor-like protein	100			
ALPV-040	44520-42745	SWPV2-036	591	591	SWPV2-036 ankyrin repeat protein	99.7	23.2	B4R	
ALPV-041	45885-44593	SWPV2-037	430	430	SWPV2-037 ankyrin repeat protein	100	27.7	B18R	
ALPV-042	47751-45934	SWPV2-038	605	605	SWPV2-038 ankyrin repeat protein	100	21.6	B4R	
ALPV-043	48462-47857	SWPV2-039	201	201	SWPV2-039 conserved hypothetical protein	100			
ALPV-044	49946-48504	SWPV2-040	480	480	SWPV2-040 ankyrin repeat protein	100	25.2	B18R	
ALPV-045	50212-51210	SWPV2-041	332	332	SWPV2-041 G protein-coupled receptor-like protein	100-			
ALPV-046	52589-51237	SWPV2-042	450	450	SWPV2-042 ankyrin repeat protein	100	29.7	VACV-Cop-B4R	
ALPV-047	53030-52656	SWPV2-043	124	124	SWPV2-043 conserved hypothetical protein	100			
ALPV-048	55603-53198	SWPV2-044	801	801	SWPV2-044 alkaline phosphodiesterase-like protein	100			
ALPV-049	56143-55691	SWPV2-045	150	150	SWPV2-045 hypothetical protein	100			
ALPV-050	57255-56197	SWPV2-046	352	352	SWPV2-046 ankyrin repeat protein	100			
ALPV-051	58528-57302	SWPV2-047	408	408	SWPV2-047 DNase II-like protein	100			
ALPV-052	59069-58554	SWPV2-048	171	171	SWPV2-048 C-type lectin-like protein	100			
ALPV-053	59689-59249	SWPV2-049	146	146	SWPV2-049 conserved hypothetical protein	100			
ALPV-054	60104-59682	SWPV2-050	140	140	SWPV2-050 conserved hypothetical protein	100			
ALPV-055	60647-60156	SWPV2-051	163	163	SWPV2-051 conserved hypothetical protein	100			
ALPV-056	61081-60644	SWPV2-052	145	145	SWPV2-052 CNLV056 dUTPase	100	52.8	F2L	
ALPV-057	62028-61108	SWPV2-053	306	306	SWPV2-053 putative serpin	100			
ALPV-058	62601-62059	SWPV2-054	180	180	SWPV2-054 bcl-2 like protein	100			
ALPV-059	63674-62658	SWPV2-055	338	338	SWPV2-055 putative serpin	100			
ALPV-060	64555-63743	SWPV2-056	270	206	SWPV2-056 conserved hypothetical protein	99.5			
ALPV-061	66342-64645	SWPV2-057	565	565	SWPV2-057 DNA ligase	100	46.9	A50R	
ALPV-062	67433-66381	SWPV2-058	350	350	SWPV2-058 putative serpin	100	25.0	K2L	
ALPV-063	68580-67504	SWPV2-059	358	358	SWPV2-059 hydroxysteroid dehydrogenase-like protein	100	38.2	A44L	
ALPV-064	69493-68642	SWPV2-060	283	283	SWPV2-060 TGF-beta-like protein	100			
ALPV-065	71324-69573	SWPV2-061	583	583	SWPV2-061 semaphorin-like protein	100	31.7	A39R	
*ALPV-066*	*71606-71424*	*SWPV2-062*	*60*	*399*	*SWPV2-062 hypothetical protein*	*100*			
ALPV-067	71608-71784		59						hypothetical protein, unique to ALPV
*ALPV-068*	*72000-71728*	*SWPV2-062*	*90*	*399*	*SWPV2-062 hypothetical protein*	*90.8*			
ALPV-069	72255-72082	SWPV2-063	57	57	SWPV2-063 hypothetical protein	100			
ALPV-070	72415-73188	SWPV2-064	257	257	SWPV2-064 GNS1/SUR4-like protein	100			
ALPV-071	73281-73748	SWPV2-065	155	155	SWPV2-065 late transcription factor VLTF-2	100	46.6	A1L	
ALPV-072	73765-75420	SWPV2-066	551	551	SWPV2-066 putative rifampicin resistance protein, IMV assembly	100	56.2	D13L	
ALPV-073	75452-76321	SWPV2-067	289	289	SWPV2-067 mRNA capping enzyme small subunit	100	57.5	D12L	
ALPV-074	76342-77283	SWPV2-068	313	132	SWPV2-068 CC chemokine-like protein	100			
ALPV-075	77690-77361	SWPV2-069	109	109	SWPV2-069 hypothetical protein	100			
ALPV-076	77761-79668	SWPV2-070	635	635	SWPV2-070 NPH-I, transcription termination factor	100	60.8	D11L	
ALPV-077	80351-79665	SWPV2-071	228	228	SWPV2-071 mutT motif putative gene expression regulator	100	38.0	D10R	
ALPV-078	81048-80335	SWPV2-072	237	232	SWPV2-072 mutT motif	97.9	46.3	D9R	
ALPV-079	81601-81287		104			100			CNPV-077 hypothetical protein
ALPV-080	81796-82065		89						hypothetical protein, unique to ALPV
ALPV-081	82693-82448		81						hypothetical protein, unique to ALPV
ALPV-082	83172-82690	SWPV2-073	160	160	SWPV2-073 RNA polymerase subunit RPO18	100	57.5	D7R	
ALPV-083	84332-83508	SWPV2-074	274	274	SWPV2-074 Ig-like domain protein	100			
ALPV-084	86356-84455	SWPV2-075	633	633	SWPV2-075 early transcription factor small subunit VETFS	100	72.2	D6R	
ALPV-085	87561-86560	SWPV2-076	333	334	SWPV2-076 Ig-like domain protein	98.8			
ALPV-086	90271-87887	SWPV2-077	794	794	SWPV2-077 NTPase, DNA replication	100	54.3	D5R	
ALPV-087	91091-90426	SWPV2-078	221	221	SWPV2-078 CC chemokine-like protein	100			
ALPV-088	91830-91174	SWPV2-079	218	218	SWPV2-079 uracil DNA glycosylase	99.5	54.2	D4R	
*ALPV-089*	*92626-91871*	*SWPV2-080*	*251*	*303*	*SWPV2-080 putative RNA phosphatase*	*94.7*	*34.1*	*H1L*	
ALPV-090	93861-92674		395			71.9			SWPV1-075 conserved hypothetical protein
ALPV-091	93946-94311	SWPV2-081	121	112	SWPV2-081 TNFR-like protein	80.8	33.9	B28R	
ALPV-092	96471-96866	SWPV2-082	131	131	SWPV2-082 putative glutathione peroxidase	100			
ALPV-093	96891-97193	SWPV2-083	100	100	SWPV2-083 conserved hypothetical protein	100			
ALPV-094	97677-97198	SWPV2-084	159	159	SWPV2-084 conserved hypothetical protein	100			
ALPV-095	98047-97664	SWPV2-085	127	127	SWPV2-085 conserved hypothetical protein	100			
ALPV-096	98384-98133	SWPV2-086	83	83	SWPV2-086 HT motif protein	100			
*ALPV-097*	*99122-98736*	*SWPV2-087*	*128*	*146*	*SWPV2-087 conserved hypothetical protein*	*87.7*			
ALPV-098	100027-99224	SWPV2-088	267	267	SWPV2-088 virion protein	100			
ALPV-099	100102-100929	SWPV2-089	275	275	SWPV2-089 T10-like protein	100			
ALPV-100	101074-100937	SWPV2-090	45	45	SWPV2-090 conserved hypothetical protein	100			
ALPV-101	101313-101056	SWPV2-091	85	85	SWPV2-091 ubiquitin	100			
*ALPV-102*	*102422-101445*	*SWPV2-092*	*325*	*339*	*SWPV2-092 conserved hypothetical protein*	*95.9*			
ALPV-103	102692-102450	SWPV2-093	80	80	SWPV2-093 hypothetical protein	100			
ALPV-104	103285-102698	SWPV2-094	195	195	SWPV2-094 beta-NGF-like protein	100			
ALPV-105	103815-103309	SWPV2-095	168	168	SWPV2-095 putative interleukin binding protein	100			
ALPV-106	104127-103870	SWPV2-096	85	85	SWPV2-096 hypothetical protein	100			
ALPV-107	104455-104138	SWPV2-097	105	105	SWPV2-097 conserved hypothetical protein	100			
ALPV-108	105044-104472	SWPV2-098	190	190	SWPV2-098 N1R/p28-like protein	100			
ALPV-109	105246-105623	SWPV2-099	125	125	SWPV2-099 putative glutaredoxin 2, virion morphogenesis	100	32.2	G4L	
ALPV-110	106270-105566	SWPV2-100	234	234	SWPV2-100 putative elongation factor	100			
ALPV-111	106264-106572	SWPV2-101	102	102	SWPV2-101 conserved hypothetical protein	100			
ALPV-112	106708-106941	SWPV2-102	77	77	SWPV2-102 hypothetical protein	100			
ALPV-113	107186-109084	SWPV2-103	632	632	SWPV2-103 putative metalloprotease, virion morphogenesis	100	43.9	G1L	
ALPV-114	111113-109068	SWPV2-104	681	681	SWPV2-104 NPH-II, RNA helicase	100	43.8	I8R	
ALPV-115	111148-112416	SWPV2-105	422	422	SWPV2-105 virion core proteinase	100	54.4	I7L	
ALPV-116	112421-113596	SWPV2-106	391	391	SWPV2-106 DNA-binding protein	100	34.8	I6L	
ALPV-117	113597-113842	SWPV2-107	81	81	SWPV2-107 putative IMV membrane protein	100			
ALPV-118	113864-114403	SWPV2-108	179	179	SWPV2-108 thymidine kinase	100	52.0	J2R	
ALPV-119	114524-114772	SWPV2-109	82	82	SWPV2-109 HT motif protein	100			
ALPV-120	114842-115711	SWPV2-110	289	289	SWPV2-110 DNA-binding phosphoprotein	99.7	33.5	I3L	
ALPV-121	115712-115921	SWPV2-111	69	69	SWPV2-111 conserved hypothetical protein	100			
ALPV-122	115928-116860	SWPV2-112	310	310	SWPV2-112 DNA-binding virion protein	100	58.0	I1L	
ALPV-123	117040-118998	SWPV2-113	652	652	SWPV2-113 conserved hypothetical protein	100	20.9	O1L	
ALPV-124	118928-119323	SWPV2-114	131	131	SWPV2-114 virion core protein	100	34.98	E11L	
ALPV-125	119601-119320	SWPV2-115	93	93	SWPV2-115 putative IMV redox protein, virus assembly	100	51.6	E10R	
ALPV-126	119628-122594	SWPV2-116	988	988	SWPV2-116 DNA polymerase	100	50.3	E9L	
ALPV-127	123413-122586		275			80.4	48.2	E8R	SWPV1-111 putative membrane protein
ALPV-128	125130-123415	SWPV2-117	571	502	SWPV2-117 conserved hypothetical protein	87.2	49.9	E6R	
ALPV-129	130930-125192	SWPV2-118	1912	1916	SWPV2-118 variola B22R-like protein	99.8			
*ALPV-130*	*136264-130997*	*SWPV2-119*	*1755*	*1767*	*SWPV2-119 variola B22R-like protein*	*99.3*			
*ALPV-131*	*142243-136544*	*SWPV2-120*	*1899*	*1839*	*SWPV2-120 variola B22R-like protein*	*95.5*			
ALPV-132	142444-142992	SWPV2-122	182	182	SWPV2-122 RNA polymerase subunit RPO30	100	57.0	E4L	
ALPV-133	143024-145189	SWPV2-123	721	721	SWPV2-123 conserved hypothetical protein	100	28.0	E2L	
ALPV-134	145182-146600	SWPV2-124	472	472	SWPV2-124 poly(A) polymerase large subunit PAPL	100	50.5	E1L	
ALPV-135	146953-146594	SWPV2-125	119	119	SWPV2-125 DNA-binding virion core protein	100	38.8	F17R	
ALPV-136	147029-147652	SWPV2-126	207	207	SWPV2-126 conserved hypothetical protein	100			
ALPV-137	147746-148192	SWPV2-127	148	148	SWPV2-127 conserved hypothetical protein	100	40.4	F15L	
ALPV-138	148426-148725	SWPV2-128	99	99	SWPV2-128 conserved hypothetical protein	100			
ALPV-139	154234-148796	SWPV2-129	1812	1801	SWPV2-129 variola B22R-like protein	99.4			
ALPV-140	154384-155520	SWPV2-130	378	378	SWPV2-130 putative palmitoylated EEV envelope lipase	100	38.0	F13L	
ALPV-141	155598-157475	SWPV2-131	625	625	SWPV2-131 putative EEV maturation protein	100	26.3	F12L	
ALPV-142	157518-158906	SWPV2-132	462	462	SWPV2-132 conserved hypothetical protein	100	27.0	F11L	
ALPV-143	158997-160331	SWPV2-133	444	444	SWPV2-133 putative serine/threonine protein kinase, virus assembly	100	53.6	F10L	
ALPV-144	160306-160947	SWPV2-134	213	213	SWPV2-134 conserved hypothetical protein	100	31.5	F9L	
ALPV-145	161030-161230	SWPV2-135	66	66	SWPV2-135 conserved hypothetical protein	100			
ALPV-146	161556-162110	SWPV2-136	184	184	SWPV2-136 HAL3-like domain protein	100			
ALPV-147	162371-163336	SWPV2-137	321	321	SWPV2-137 N1R/p28-like protein	100			
ALPV-148	163448-165463	SWPV2-138	671	671	SWPV2-138 ankyrin repeat protein	100	26.0	M1L	
ALPV-149	165489-167159	SWPV2-139	556	556	SWPV2-139 ankyrin repeat protein	100	26.1	B4R	
ALPV-150	167380-168702	SWPV2-140	440	440	SWPV2-140 conserved hypothetical protein	100	33.3	G5R	
ALPV-151	168710-168898	SWPV2-141	62	62	SWPV2-141 RNA polymerase subunit RPO7	98.4	55.2	G5.5R	
ALPV-152	168891-169457	SWPV2-142	188	188	SWPV2-142 conserved hypothetical protein	100	31.8	G6R	
ALPV-153	170468-169422	SWPV2-143	348	348	SWPV2-143 virion core protein	100	34.6	G7L	
ALPV-154	171554-170634	SWPV2-144	306	306	SWPV2-144 putative thioredoxin binding protein	100			
ALPV-155	171682-171915		77			97.1			CNPV-150 ankyrin repeat protein
ALPV-156	173269-172031	SWPV2-145	412	412	SWPV2-145 ankyrin repeat protein	100	42.9	M1L	
ALPV-157	173945-173496	SWPV2-146	149	149	SWPV2-146 hypothetical protein	100			
ALPV-158	175111-174173	SWPV2-147	312	312	SWPV2-147 Rep-like protein	100			
ALPV-159	181355-175545	SWPV2-148	1936	875	SWPV2-148 variola B22R-like protein	98.2			
*ALPV-160*	*186885-181408*	*SWPV2-149*	*1825*	*1831*	*SWPV2-149 variola B22R-like protein*	*99.7*			
*ALPV-161*	*187202-189685*	*SWPV2-150*	*827*	*834*	*SWPV2-150 hypothetical protein*	*90.5*			
ALPV-162	190813-189782	SWPV2-151	343	343	SWPV2-151 TGF-beta-like protein	100			
ALPV-163	190815-191294		159			64.7			CNPV-157 TGF-beta-like protein
*ALPV-164*	*191763-192263*	*SWPV2-150*	*166*	*834*	*SWPV2-150 hypothetical protein*	*47.8*			
ALPV-165	192733-193446		237			97.2			CNPV-159 N1R/p28-like protein
ALPV-166	193490-194494		334			91.3			CNPV-169 N1R/p28-like protein
ALPV-167	194549-195412		287			78.7			CNPV-169 N1R/p28-like protein
ALPV-168	195417-195821		134			98.5			CNPV-160 N1R/p28-like protein
ALPV-169	197008-195920	SWPV2-152	362	358	SWPV2-152 TGF-beta-like protein	98.9			
ALPV-170	197058-197507	SWPV2-153	149	149	SWPV2-153 TGF-beta-like protein	100			
ALPV-171	197843-198883	SWPV2-154	346	320	SWPV2-154 N1R/p28-like protein	95.3			
ALPV-172	199118-200155	SWPV2-155	345	345	SWPV2-155 Ig-like domain protein	99.7			
ALPV-173	200427-200933	SWPV2-156	168	168	SWPV2-156 Ig-like domain protein	97			
*ALPV-174*	*201028-202059*	*SWPV2-157*	*343*	*350*	*SWPV2-157 N1R/p28-like protein*	*93.6*			
ALPV-175	202011-202415		134			92.5			CNPV-226 N1R/p28-like protein
ALPV-176	202488-203126	SWPV2-158	212	212	SWPV2-158 thymidylate kinase	100	45.2	A48R	
ALPV-177	203179-203961	SWPV2-159	260	260	SWPV2-159 late transcription factor VLTF-1	100	66.2	G8R	
ALPV-178	203975-204982	SWPV2-160	335	335	SWPV2-160 putative myristylated protein	100	38.0	G9R	
ALPV-179	204983-205714	SWPV2-161	243	243	SWPV2-161 putative myristylated IMV envelope protein	100	54.7	L1R	
ALPV-180	205774-206064	SWPV2-162	96	96	SWPV2-162 conserved hypothetical protein	100			
ALPV-181	206965-206054	SWPV2-163	303	303	SWPV2-163 conserved hypothetical protein	100	42.0	L3L	
ALPV-182	206991-207749	SWPV2-164	252	252	SWPV2-164 DNA-binding virion core protein	100	36.3	L4R	
ALPV-183	207750-208142	SWPV2-165	130	130	SWPV2-165 conserved hypothetical protein	100	41.6	L5R	
ALPV-184	208096-208542	SWPV2-166	148	148	SWPV2-166 putative IMV membrane protein	100	42.5	J1R	
ALPV-185	208576-209484	SWPV2-167	302	302	SWPV2-167 poly(A) polymerase small subunit PAPS	100	56.0	J3R	
ALPV-186	209481-210041	SWPV2-168	186	186	SWPV2-168 RNA polymerase subunit RPO22	100	55.0	J4R	
ALPV-187	210444-210034	SWPV2-169	136	136	SWPV2-169 conserved hypothetical protein	100	47.9	J5L	
ALPV-188	210487-214353	SWPV2-170	1288	1288	SWPV2-170 RNA polymerase subunit RPO147	100	70.6	J6R	
ALPV-189	214856-214356	SWPV2-171	166	166	SWPV2-171 putative protein-tyrosine phosphatase, virus assembly	100	47.6	H1L	
ALPV-190	214872-215441	SWPV2-172	189	189	SWPV2-172 conserved hypothetical protein	100	48.9	H2R	
ALPV-191	216503-215517	SWPV2-173	328	328	SWPV2-173 ankyrin repeat protein	100			
ALPV-192	217538-216546	SWPV2-174	330	330	SWPV2-174 putative IMV envelope protein	100	32.2	H3L	
ALPV-193	220029-217630	SWPV2-175	799	799	SWPV2-175 RNA polymerase associated protein RAP94	100	55.2	H4L	
ALPV-194	220198-220710	SWPV2-176	170	170	SWPV2-176 late transcription factor VLTF-4	100			
ALPV-195	220711-221661	SWPV2-177	316	316	SWPV2-177 DNA topoisomerase	100	58.0	H6R	
ALPV-196	221666-222127	SWPV2-178	153	153	SWPV2-178 conserved hypothetical protein	100	33.8	H7R	
ALPV-197	222401-222090	SWPV2-179	103	103	SWPV2-179 conserved hypothetical protein	100			
ALPV-198	222409-224949	SWPV2-180	846	846	SWPV2-180 mRNA capping enzyme large subunit	100	53.9	D1R	
ALPV-199	225020-225340	SWPV2-181	106	106	SWPV2-181 HT motif protein	100			
ALPV-200	225759-225337	SWPV2-182	140	140	SWPV2-182 virion protein	100			
ALPV-201	225813-226247	SWPV2-183	144	144	SWPV2-183 hypothetical protein	100			
ALPV-202	226312-226884	SWPV2-184	190	190	SWPV2-184 conserved hypothetical protein	100			
ALPV-203	226950-227777	SWPV2-185	275	275	SWPV2-185 N1R/p28-like protein	100			
ALPV-204	228314-227844	SWPV2-186	156	156	SWPV2-186 C-type lectin-like protein	100	30.8	A34R	
ALPV-205	228622-229299	SWPV2-187	225	225	SWPV2-187 deoxycytidine kinase-like protein	100			
ALPV-206	229305-229805	SWPV2-188	166	166	SWPV2-188 Rep-like protein	99.4			
ALPV-207	229864-230367	SWPV2-189	167	167	SWPV2-189 conserved hypothetical protein	100			
ALPV-208	230421-231251	SWPV2-190	276	276	SWPV2-190 N1R/p28-like protein	100			
ALPV-209	231324-232472	SWPV2-191	382	382	SWPV2-191 N1R/p28-like protein	100			
ALPV-210	232528-232713	SWPV2-192	61	61	SWPV2-192 conserved hypothetical protein	100			
ALPV-211	232932-233888	SWPV2-193	318	318	SWPV2-193 N1R/p28-like protein	100			
ALPV-212	233949-235367	SWPV2-194	472	472	SWPV2-194 putative photolyase	100			
ALPV-213	235424-235579		51						hypothetical protein, unique to ALPV, containing a transmembrane helix
ALPV-214	235557-236078	SWPV2-195	173	173	SWPV2-195 N1R/p28-like protein	100			
*ALPV-215*	*236184-236723*	*SWPV2-196*	*179*	*200*	*SWPV2-196 conserved hypothetical protein*	*89*			
ALPV-216	236767-237699	SWPV2-197	310	310	SWPV2-197 N1R/p28-like protein	100			
ALPV-217	237747-238142	SWPV2-198	131	131	SWPV2-198 N1R/p28-like protein	100			
ALPV-218	238197-238361	SWPV2-199	54	54	SWPV2-199 conserved hypothetical protein	100			
ALPV-219	238421-238951	SWPV2-200	176	176	SWPV2-200 N1R/p28-like protein	100			
ALPV-220	239662-239012	SWPV2-201	216	216	SWPV2-201 deoxycytidine kinase-like protein	100			
ALPV-221	239836-240906	SWPV2-202	356	356	SWPV2-202 vaccinia C4L/C10L-like protein	100	28.1	C10L	
ALPV-222	241181-241795	SWPV2-203	204	204	SWPV2-203 CC chemokine-like protein	100			
ALPV-223	241885-243090	SWPV2-204	401	401	SWPV2-204 conserved hypothetical protein	100			
ALPV-224	243204-244196	SWPV2-205	330	330	SWPV2-205 N1R/p28-like protein	100			
ALPV-225	244284-245555	SWPV2-206	423	223	SWPV2-206 N1R/p28-like protein	99.5			
ALPV-226	245795-245619		58						hypothetical protein, unique to ALPV, containing a transmembrane helix
ALPV-227	246013-245858		51						hypothetical protein, unique to ALPV, containing a transmembrane helix
ALPV-228	246363-247412	SWPV2-207	349	349	SWPV2-207 N1R/p28-like protein	100			
ALPV-229	247466-248308		280			80.9			SWPV1-198 N1R/p28-like protein
*ALPV-230*	*248847-248644*	*SWPV2-208*	*67*	*85*	*SWPV2-208 N1R/p28-like protein*	*62.7*			
*ALPV-231*	*249295-249483*	*SWPV2-209*	*62*	*213*	*SWPV2-209 N1R/p28-like protein*	*100*			
*ALPV-232*	*250029-250292*	*SWPV2-210*	*87*	*285*	*SWPV2-210 N1R/p28-like protein*	*100*			
ALPV-233	250283-250615		110			98.2			CNPV-227 N1R/p28-like protein
ALPV-234	253677-251134	SWPV2-211	847	847	SWPV2-211 ankyrin repeat protein	99.9	31.6	B4R	
ALPV-235	253931-254650	SWPV2-212	239	239	SWPV2-212 hypothetical protein	100			
ALPV-236	254650-255027		125			73.3			CNPV-227 N1R/p28-like protein
*ALPV-237*	*255062-255346*	*SWPV2-214*	*94*	*126*	*SWPV2-214 N1R/p28-like protein*	*96.4*			
ALPV-238	257103-255799	SWPV2-215	434	434	SWPV2-215 ankyrin repeat protein	100	27.3	B4R	
ALPV-239	257301-257498	SWPV2-216	65	65	SWPV2-216 hypothetical protein	100			
ALPV-240	257446-257922	SWPV2-217	158	158	SWPV2-217 MyD116-like domain protein	100			
ALPV-241	257952-258566	SWPV2-218	204	204	SWPV2-218 CC chemokine-like protein	98			
ALPV-242	258748-260163	SWPV2-219	471	471	SWPV2-219 ankyrin repeat protein	100	31.4	M1L	
ALPV-243	260183-261709	SWPV2-220	508	508	SWPV2-220 ankyrin repeat protein	100	37.0	M1L	
ALPV-244	261780-263084	SWPV2-221	434	432	SWPV2-221 conserved hypothetical protein	99.5			
ALPV-245	263129-264100	SWPV2-222	323	323	SWPV2-222 ribonucleotide reductase small subunit	100	70.9	F4L	
ALPV-246	264281-265606	SWPV2-223	441	441	SWPV2-223 ankyrin repeat protein	99.8	33.3	B4R	
ALPV-247	266342-265665	SWPV2-224	225	225	SWPV2-224 late transcription factor VLTF-3	100	76.7	A2L	
ALPV-248	266557-266330	SWPV2-225	75	75	SWPV2-225 virion redox protein	100			
ALPV-249	268550-266571	SWPV2-226	659	659	SWPV2-226 virion core protein P4b	100	54.1	A3L	
ALPV-250	269284-268637	SWPV2-227	215	215	SWPV2-227 immunodominant virion protein	100			
ALPV-251	269323-269832	SWPV2-228	169	169	SWPV2-228 RNA polymerase subunit RPO19	100	55.2	A5R	
ALPV-252	270948-269827	SWPV2-229	373	373	SWPV2-229 conserved hypothetical protein	100	39.3	A6L	
ALPV-253	273084-270955	SWPV2-230	709	709	SWPV2-230 early transcription factor large subunit VETFL	100	59.6	A7L	
ALPV-254	273148-274050	SWPV2-231	300	300	SWPV2-231 intermediate transcription factor VITF-3	100	38.7	A8R	
ALPV-255	274242-274015	SWPV2-232	75	75	SWPV2-232 putative IMV membrane protein	100			
ALPV-256	276924-274243	SWPV2-233	893	893	SWPV2-233 virion core protein P4a	100	39.6	A10L	
ALPV-257	276942-277781	SWPV2-234	279	279	SWPV2-234 conserved hypothetical protein	100	39.5	A11R	
ALPV-258	278284-277778	SWPV2-235	168	168	SWPV2-235 virion protein	99.4	32.9	A12L	
ALPV-259	278299-278556	SWPV2-236	85	56	SWPV2-236 conserved hypothetical protein	87.0			
ALPV-260	278754-278545	SWPV2-237	69	69	SWPV2-237 putative IMV membrane protein	100			
ALPV-261	279080-278802	SWPV2-238	92	92	SWPV2-238 putative IMV membrane protein	100	27.5	A14L	
ALPV-262	279258-279097	SWPV2-239	53	53	SWPV2-239 putative IMV membrane virulence factor	100	35.3	A 14.5L	
ALPV-263	279564-279274	SWPV2-240	96	96	SWPV2-240 conserved hypothetical protein	100			
ALPV-264	280654-279548	SWPV2-241	368	368	SWPV2-241 predicted myristylated protein	100	43.0	A16L	
ALPV-265	281248-280670	SWPV2-242	192	192	SWPV2-242 putative phosphorylated IMV membrane protein	100	33.2	A17L	
ALPV-266	281266-282654	SWPV2-243	462	462	SWPV2-243 DNA helicase, transcriptional elongation	100	50.2	A18R	
ALPV-267	282891-282622	SWPV2-244	89	89	SWPV2-244 conserved hypothetical protein	100	42.9	A19L	
ALPV-268	283237-282899	SWPV2-245	112	112	SWPV2-245 conserved hypothetical protein	100	45.3	A21L	
ALPV-269	283236-284540	SWPV2-246	434	434	SWPV2-246 DNA polymerase processivity factor	100	25.9	A20R	
ALPV-270	284537-284995	SWPV2-247	152	152	SWPV2-247 Holliday junction resolvase protein	100	45.0	A22R	
ALPV-271	285012-286163	SWPV2-248	383	383	SWPV2-248 intermediate transcription factor VITF-3	100	51.3	A23R	
ALPV-272	286189-289662	SWPV2-249	1157	1157	SWPV2-249 RNA polymerase subunit RPO132	100	74.6	A24R	
ALPV-273	291456-289651	SWPV2-250	601	601	SWPV2-250 A type inclusion-like protein	100			
ALPV-274	292918-291491	SWPV2-251	475	475	SWPV2-251 A type inclusion-like/fusion protein	100	56.4	A27L	
ALPV-275	293341-292919	SWPV2-252	140	140	SWPV2-252 conserved hypothetical protein	100	41.8	A28L	
ALPV-276	294263-293346	SWPV2-253	305	305	SWPV2-253 RNA polymerase subunit RPO35	100	42.6	A29L	
ALPV-277	294465-294238	SWPV2-254	75	75	SWPV2-254 conserved hypothetical protein	100			
ALPV-278	294590-294931	SWPV2-255	113	113	SWPV2-255 conserved hypothetical protein	100	31.6	A31R	
ALPV-279	294940-295302	SWPV2-256	120	120	SWPV2-256 conserved hypothetical protein	100			
ALPV-280	296145-295291	SWPV2-257	284	284	SWPV2-257 DNA packaging protein	100	47.1	A32L	
ALPV-281	296260-296805	SWPV2-258	181	181	SWPV2-258 C-type lectin-like EEV protein	100			
ALPV-282	297030-297854	SWPV2-259	274	274	SWPV2-259 conserved hypothetical protein	100			
ALPV-283	297914-298723	SWPV2-260	269	269	SWPV2-260 putative tyrosine protein kinase	100			
ALPV-284	298766-299782	SWPV2-261	338	338	SWPV2-261 putative serpin	100			
ALPV-285	300562-299804	SWPV2-262	252	252	SWPV2-262 conserved hypothetical protein	100			
ALPV-286	300672-301604	SWPV2-263	310	310	SWPV2-263 G protein-coupled receptor-like protein	100			
ALPV-287	301615-301905	SWPV2-264	96	96	SWPV2-264 conserved hypothetical protein	100			
ALPV-288	301971-302501	SWPV2-265	176	169	SWPV2-265 beta-NGF-like protein	96			
ALPV-289	302911-302519	SWPV2-266	130	130	SWPV2-266 HT motif protein	100			
ALPV-290	303015-303659	SWPV2-267	214	214	SWPV2-267 conserved hypothetical protein	100			
ALPV-291	304032-303670	SWPV2-268	120	120	SWPV2-268 HT motif protein	100			
ALPV-292	304198-304533	SWPV2-269	111	111	SWPV2-269 CC chemokine-like protein	100			
ALPV-293	304612-305193	SWPV2-270	193	193	SWPV2-270 putative interleukin binding protein	100			
ALPV-294	305303-305683	SWPV2-271	126	126	SWPV2-271 EGF-like protein	100	34.8	C11R	
ALPV-295	305685-306602	SWPV2-272	305	305	SWPV2-272 putative serine/threonine protein kinase	100	41.1	B1R	
ALPV-296	306645-307127	SWPV2-273	160	160	SWPV2-273 conserved hypothetical protein	100			
ALPV-297	307210-307677	SWPV2-274	155	147	SWPV2-274 C-type lectin-like protein	99.3	22.6	A34R	
ALPV-298	307720-308139	SWPV2-275	139	139	SWPV2-275 putative interleukin binding protein	100			
ALPV-299	308208-308435	SWPV2-276	75	75	SWPV2-276 conserved hypothetical protein	100			
ALPV-300	308637-310421	SWPV2-277	594	594	SWPV2-277 ankyrin repeat protein	99.8	22.3	B4R	
ALPV-301	310445-310669	SWPV2-278	74	74	SWPV2-278 hypothetical protein	100			
ALPV-302	310712-311566	SWPV2-279	284	284	SWPV2-279 ankyrin repeat protein	99.6	33.9	B24R	
ALPV-303	311621-312913	SWPV2-280	430	430	SWPV2-280 ankyrin repeat protein	99.8		C18L	
ALPV-304	313106-314296	SWPV2-281	396	396	SWPV2-281 ankyrin repeat protein	100	28.6	M1L	
ALPV-305	314299-315675	SWPV2-282	458	458	SWPV2-282 ankyrin repeat protein	100	32.5	B4R	
ALPV-306	315784-317997	SWPV2-283	737	737	SWPV2-283 ankyrin repeat protein	100	25.8	M1L	
ALPV-307	318053-319768	SWPV2-284	571	571	SWPV2-284 ankyrin repeat protein	100	28.8	B4R	
ALPV-308	319772-320674	SWPV2-285	300	300	SWPV2-285 putative serine/threonine protein kinase	100	24.6	M1L	
ALPV-309	320747-321481	SWPV2-286	244	244	SWPV2-286 ankyrin repeat protein	100	32.8	B1R	
ALPV-310	322082-323665	SWPV2-287	527	527	SWPV2-287 ankyrin repeat protein	100	23.7	M1L	
ALPV-311	324261-323680	SWPV2-288	193	193	SWPV2-288 conserved hypothetical protein	100	26.1	B18R	
ALPV-312	324329-325831	SWPV2-289	500	500	SWPV2-289 ankyrin repeat protein	100			
ALPV-313	326047-327447	SWPV2-290	466	466	SWPV2-290 ankyrin repeat protein	100	28.3	M1L	
ALPV-314	327518-328306	SWPV2-291	262	262	SWPV2-291 N1R/p28-like protein	100	28.2	B4R	
ALPV-315	328368-328586	SWPV2-292	72	72	SWPV2-292 hypothetical protein	100			
ALPV-316	329054-328590	SWPV2-293	154	154	SWPV2-293 C-type lectin-like protein	100			
ALPV-317	329231-330304	SWPV2-294	357	357	SWPV2-294 ankyrin repeat protein	100	34.4	A40R	
ALPV-318	330452-331042	SWPV2-295	196	196	SWPV2-295 ankyrin repeat protein	100			
ALPV-319	331147-332760	SWPV2-296	537	537	SWPV2-296 ankyrin repeat protein	100	37.4	M1L	
ALPV-320	332794-333168	SWPV2-297	124	124	SWPV2-297 EFc-like protein	100			
ALPV-321	333178-333678	SWPV2-298	166	166	SWPV2-298 conserved hypothetical protein	100			
ALPV-322	333750-334406	SWPV2-299	218	218	SWPV2-299 Ig-like domain protein	100			
ALPV-323	334433-336322	SWPV2-300	629	629	SWPV2-300 ankyrin repeat protein	99.8			
ALPV-324	336421-337368	SWPV2-301	315	315	SWPV2-301 G protein-coupled receptor-like protein	100	40.8	B4R	
ALPV-325	337435-339069	SWPV2-302	544	544	SWPV2-302 ankyrin repeat protein	99.8			
ALPV-326	339250-339417	SWPV2-303	55	55	SWPV2-303 hypothetical protein	100	33.9	B4R	
*ALPV-327*	*339592-341124*	*SWPV2-304*	*510*	*514*	*SWPV2-304 ankyrin repeat protein*	*99.2*			
ALPV-328	341457-343424	SWPV2-305	655	637	SWPV2-305 ankyrin repeat protein	96.6	36.8	M1L	
ALPV-329	343616-345025	SWPV2-306	469	469	SWPV2-306 Ig-like domain protein	100	25.8	M1L	
ALPV-330	345157-345531	SWPV2-307	124	124	SWPV2-307 EFc-like protein	100			
*ALPV-331*	*345865-347868*	*SWPV2-308*	*667*	*689*	*SWPV2-308 ankyrin repeat protein*	*96.5*			
*ALPV-332*	*348446-347985*	*SWPV2-309*	*153*	*186*	*SWPV2-309 conserved hypothetical protein*	*86.9*			*identical to ALPV-005*
ALPV-333	349231-348563	SWPV2-310	222	222	SWPV2-310 conserved hypothetical protein	100			identical to ALPV-004
ALPV-334	349357-349584		75			56.1			SWPV1-002 C-type lectin-like protein, identical to ALPV-003
ALPV-335	349639-350265	SWPV2-311	208	208	SWPV2-311 C-type lectin-like protein	100			identical to ALPV-002
ALPV-336	351083-350568	SWPV2-312	171	171	SWPV2-312 hypothetical protein	100			identical to ALPV-001

Note: ALPV, albatrosspox virus (GenBank accession no. MW365933); SWPV1, shearwaterpox virus 1 (GenBank accession no. KX857216); SWPV2, shearwaterpox virus 2 (GenBank accession no. KX857215); CNPV, canarypox virus (GenBank accession no. AY318871). Avipoxviruses as being identity to SWPV2 unless indicated in the note column. Truncated or fragmented ORFs of ALPV compared to SWPV2 are highlighted in italic font.

**Table 3:** Related poxvirus genome sequences used in further analysis of ALPV.

Virus	Abbreviation	GenBank Accession Number	Reference
Albatrosspox virus	ALPV	MW365933	This study
Canarypox virus	CNPV	AY318871	[32]
Cheloniidpox virus 1	ChePV-1	MT799800	[61]
Fowlpox virus	FWPV	AF198100, MF766430-32, MH709124-25, MH719203, MH734528, AJ581527, MW142017	[28,29,62,63]
Flamingopox virus	FGPV	MF678796	[24]
Magpiepox virus	MPPV	MK903864	[31]
Mudlarkpox virus	MLPV	MT978051	[30]
Nile crocodilepox virus	CRV	DQ356948	[64]
Penguinpox virus	PEPV	KJ859677	[33]
Penguinpox virus 2	PEPV2	MW296038	[19]
Pigeonpox virus	FeP2	KJ801920	[33]
Saltwater crocodilepox virus 1	SwCRV1	MG450915	[36,65]
Shearwaterpox virus 1	SWPV1	KX857216	[25]
Shearwaterpox virus 2	SWPV2	KX857215	[25]
Turkeypox virus	TKPV	NC_028238	[34]

## Data Availability

The complete genome sequence and associated datasets generated during this study were deposited in GenBank under the accession number MW365933.

## Supplementary Materials

The following are available online at https://www.mdpi.com/article/10.3390/pathogens10050575/s1, Figure S1: Maximum likelihood (ML) phylogenetic tree from partial nucleotide sequences of the DNA polymerase gene of selected avipoxviruses, Figure S2: Maximum likelihood phylogenetic tree from partial nucleotide sequences of the p4b gene of selected avipoxviruses.
