# Supplementary material for: Genomic Characterisation of a Novel Avipoxvirus Isolated from an Endangered Northern Royal Albatross (Diomedea sanfordi)
Source: Pathogens. 2021 May 9;10(5):575. doi: 10.3390/pathogens10050575 (PMC8151833; doi:10.3390/pathogens10050575)
Supplement: Supplementary file 1 [file pathogens-10-00575-s001.zip › pathogens-1202557-supplementary.pdf]

pathogens

MDPI

Supplementary File

Genomic characterisation of a novel avipoxvirus isolated from an endangered northern royal albatross (*Diomedea sanfordi*)

Subir Sarker[1]*, Ajani Athukorala[1], Tadiwa Nyandowe[1], Timothy R. Bowden[2,3] and David B. Boyle[2]

[1] Department of Physiology, Anatomy and Microbiology, School of Life Sciences, La Trobe University, Melbourne, VIC 3086, Australia. (S.S. email: S.Sarker@latrobe.edu.au; A.A. email: a.athukorala@latrobe.edu.au; T.N. email: 19918447@students.latrobe.edu.au).

[2] CSIRO Livestock Industries, Australian Animal Health Laboratory, Geelong, VIC 3220, Australia. (T.R.B. email: timothy.bowden@csiro.au; D.B.B email: davidboyle48@gmail.com).

[3] Present address: CSIRO Australian Animal Health Laboratory, Australian Centre for Disease Preparedness, Geelong, VIC 3220, Australia. (T.R.B. email: timothy.bowden@csiro.au).

* Correspondence: S.Sarker@latrobe.edu.au; Tel.: +61 3 9479 2317; fax: (+61) 3 9479 1222

pathogens

MDPI

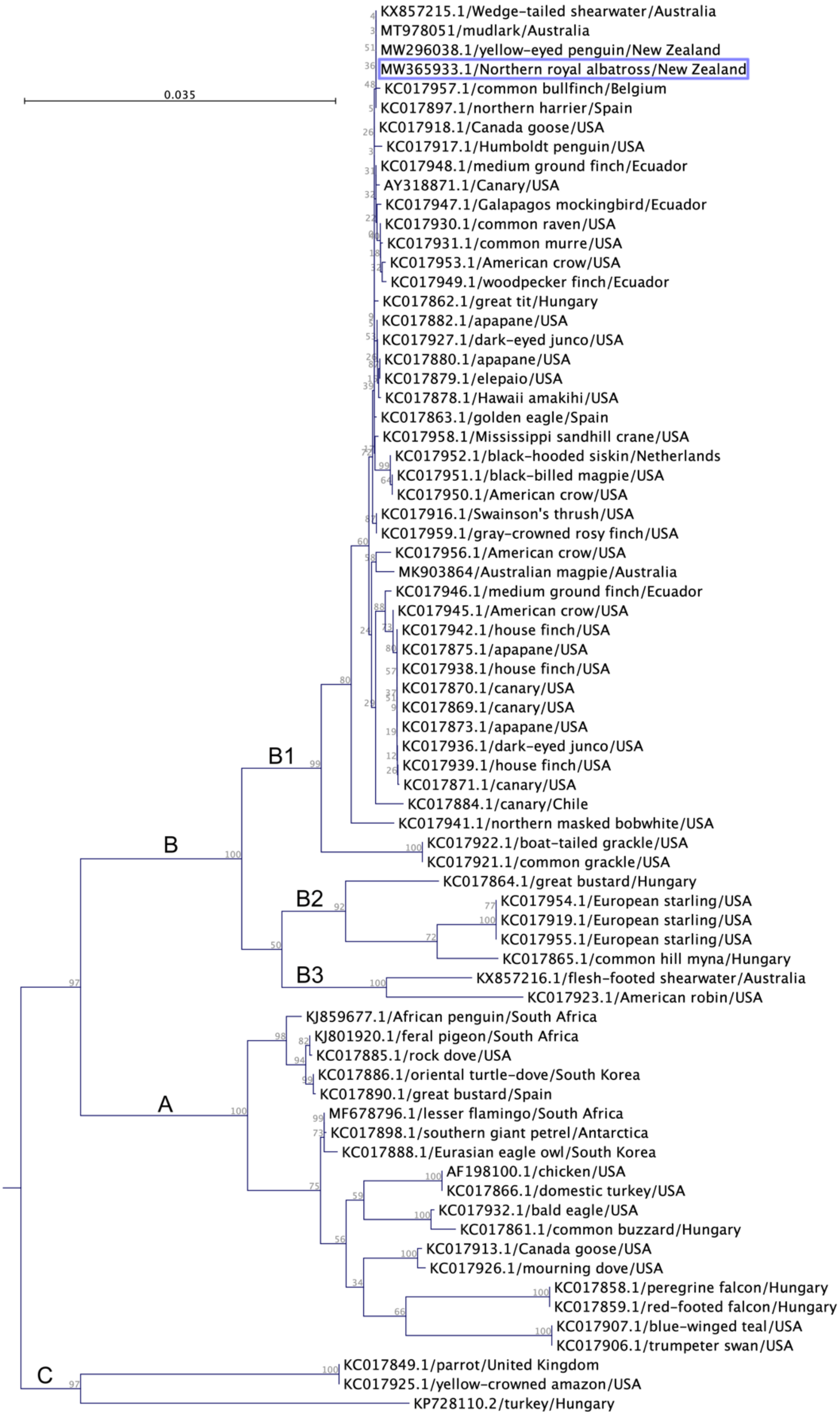

Figure S1: Maximum likelihood (ML) phylogenetic tree from partial nucleotide sequences of the DNA polymerase gene of selected avipoxviruses. Labels at branch tips refer to GenBank accession number/species/country of origin. The numbers on the left show bootstrap values as percentages and the position of ALPV is highlighted using a purple box. The ML tree is displayed as a phylogram. The bootstrap value assigned to a node in the output tree is the percentage (0-100) of the bootstrap resamples which resulted in a tree containing the same subtree as that rooted at the node. According to Gyuranecz *et al* (2013), major clades are designated A, B and C, while B1-B3 represent subclades.

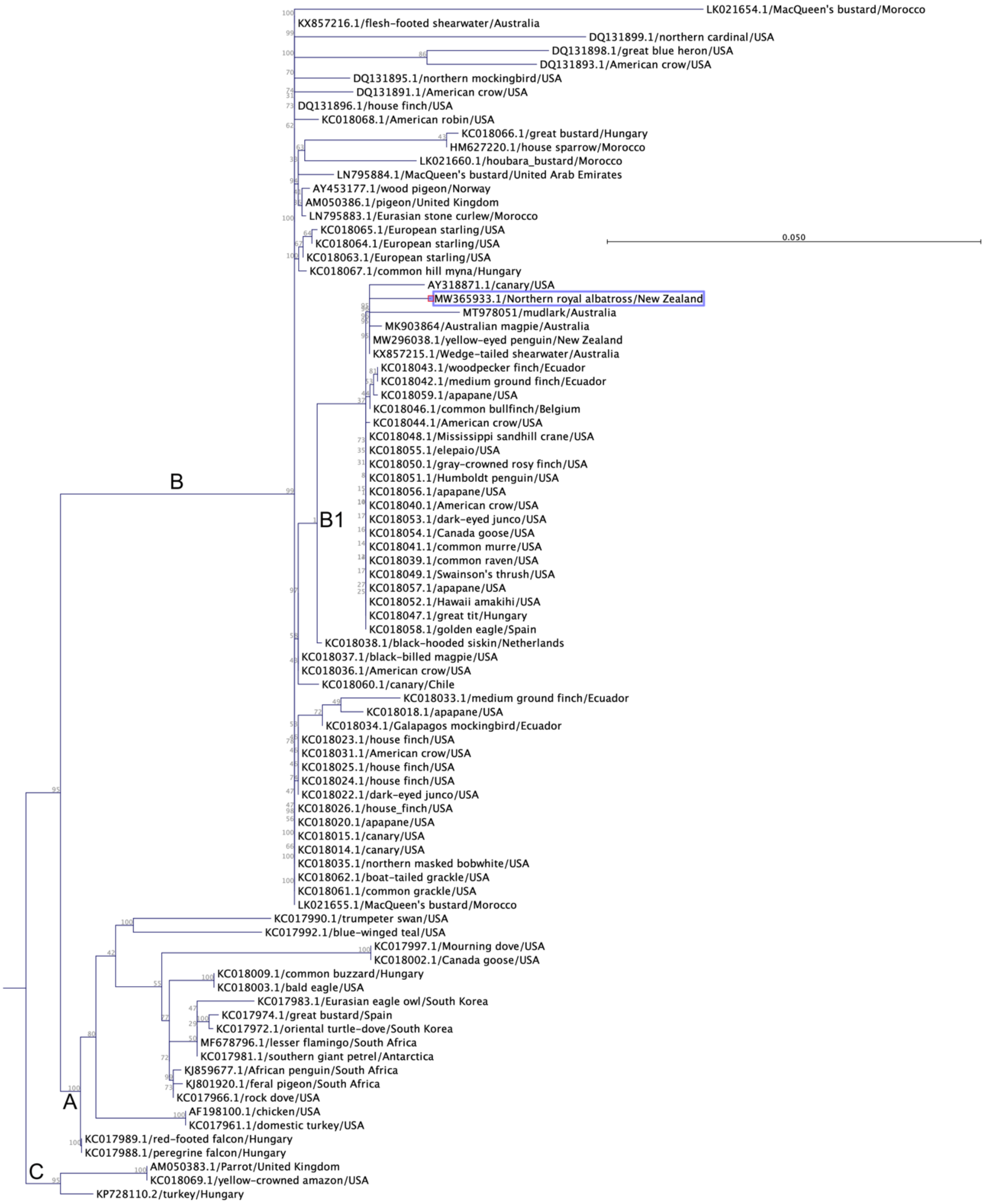

Figure S2: Maximum likelihood phylogenetic tree from partial nucleotide sequences of the p4b gene of selected avipoxviruses. Labels at branch tips refer to GenBank accession number/species/country of origin. The numbers on the left show bootstrap values as percentages and the position of ALPV is highlighted using a purple box. The ML tree is displayed as a phylogram. The bootstrap value assigned to a node in the output tree is the percentage (0-100) of the bootstrap resamples which resulted in a tree containing the same subtree as that rooted at the node. According to Gyuranecz *et al.* (2013), major clades are designated A, B and C, while B1 represents a subclade.
